# Advances in sRNA-mediated regulation of *Salmonella* infection in the host

**DOI:** 10.3389/fcimb.2025.1503337

**Published:** 2025-05-15

**Authors:** Ting He, Yonghui Ding, Yuling Sun, Tiansen Li

**Affiliations:** School of Tropical Agriculture and Forestry, Hainan University, Haikou, China

**Keywords:** *Salmonella*, sRNA, infection, virulence, regulation

## Abstract

*Salmonella* is a foodborne pathogen that enters the host’s body through contaminated food and water, leading to gastroenteritis and systemic diseases. It is a significant veterinary and human pathogen capable of infecting both humans and animals, with substantial impacts on public health, human well-being, and the economic development of the livestock and poultry farming industry. Small non-coding RNAs (sRNAs), typically 50–500 nucleotides (nt) in length, have been identified in various bacteria, including *Escherichia coli*, *Brucella*, *Pseudomonas aeruginosa*, and *Salmonella*. These sRNAs play crucial roles in regulating diverse physiological processes within bacteria. This review emphasizes recent advances in understanding how sRNAs regulate the virulence of *Salmonella* spp, such as the discovery of novel sRNAs like SaaS and new regulatory mechanisms of known sRNAs like RyhB-1/RyhB-2 and SdsR/Spot 42. It also outlines critical future directions, including exploring the multifaceted functions of sRNAs in lifestyle or infection phase transitions, fully elucidating their roles in regulating the host immune response, studying the combined actions of multiple sRNAs on host pathogenesis and expanding research to more *Salmonella* serotypes and diverse animal models. Through these efforts, this review aims to enhance our understanding of *Salmonella* sRNAs and their infection mechanisms.

## 1 Introduction

*Salmonella* is a facultative anaerobic bacterium belonging to the *Enterobacteriaceae* family and is classified as a Gram-negative bacterium. It possesses a wide array of antigens and a highly complex structure, which can be broadly categorized into three types: somatic (O) antigens, flagellar (H) antigens, and surface (Vi) antigens. To date, Over 2,600 serotypes of *Salmonella* have been identified globally (Kabir et al., 2025; Vakili et al., 2025). Most *Salmonella* strains possess flagella, except *Salmonella* Pullorum and *Salmonella* Gallinarum. *Salmonella* is a common pathogen responsible for zoonotic infections and is a leading cause of foodborne illness worldwide (Traore et al., 2024). In the intestinal tract, *Salmonella* is excreted through feces and can contaminate water and food via insects or other biological vectors. When contaminated water or food is ingested by humans or animals, the pathogens are once again excreted through feces, perpetuating a continuous cycle of transmission. According to survey statistics, *Salmonella*-related food poisoning cases rank among the highest globally, and *Salmonella* is widely distributed (Chlebicz and Śliżewska, 2018). *Salmonella* is commonly found in meat, eggs, poultry and milk. Contamination can occur due to substandard environmental conditions in food preparation areas and improper cooking practices, leading to illness in consumers. The presence of *Salmonella* poses a significant threat to food safety and the sustainable development of animal husbandry.

sRNAs are a class of non-coding RNAs ranging from 50 nt to 500 nt in length and are widely found in bacterial RNAs (Tsai et al., 2015; Meng et al., 2022). Previous studies have found that sRNAs are predominantly found in Gram-negative bacteria such as *Brucella*, *Escherichia coli*, *Pseudomonas aeruginosa*, and *Salmonella* and are involved in the regulation of several bacterial physiological processes including bacteriophage metabolism, iron stabilization, carbon and amino acid utilization, metal ion utilization, expression of outer membrane proteins, periplasmic membrane formation, stress response, community sensing, and virulence gene regulation (Fröhlich et al., 2016; Kim, 2016; Meng et al., 2022). For the past few years, the role played by sRNAs in bacterial-host interactions has been of great interest, and it has been demonstrated that sRNAs play an indispensable role in the process of regulating the mechanisms of bacterial pathogenesis. Therefore, the present study highlights the functions played by sRNAs in *Salmonella*-host interactions in recent years to provide ideas for improving host resistance to *Salmonella* infection.

## 2 Mechanisms of action of *Salmonella* infected hosts

**Figure 1:**
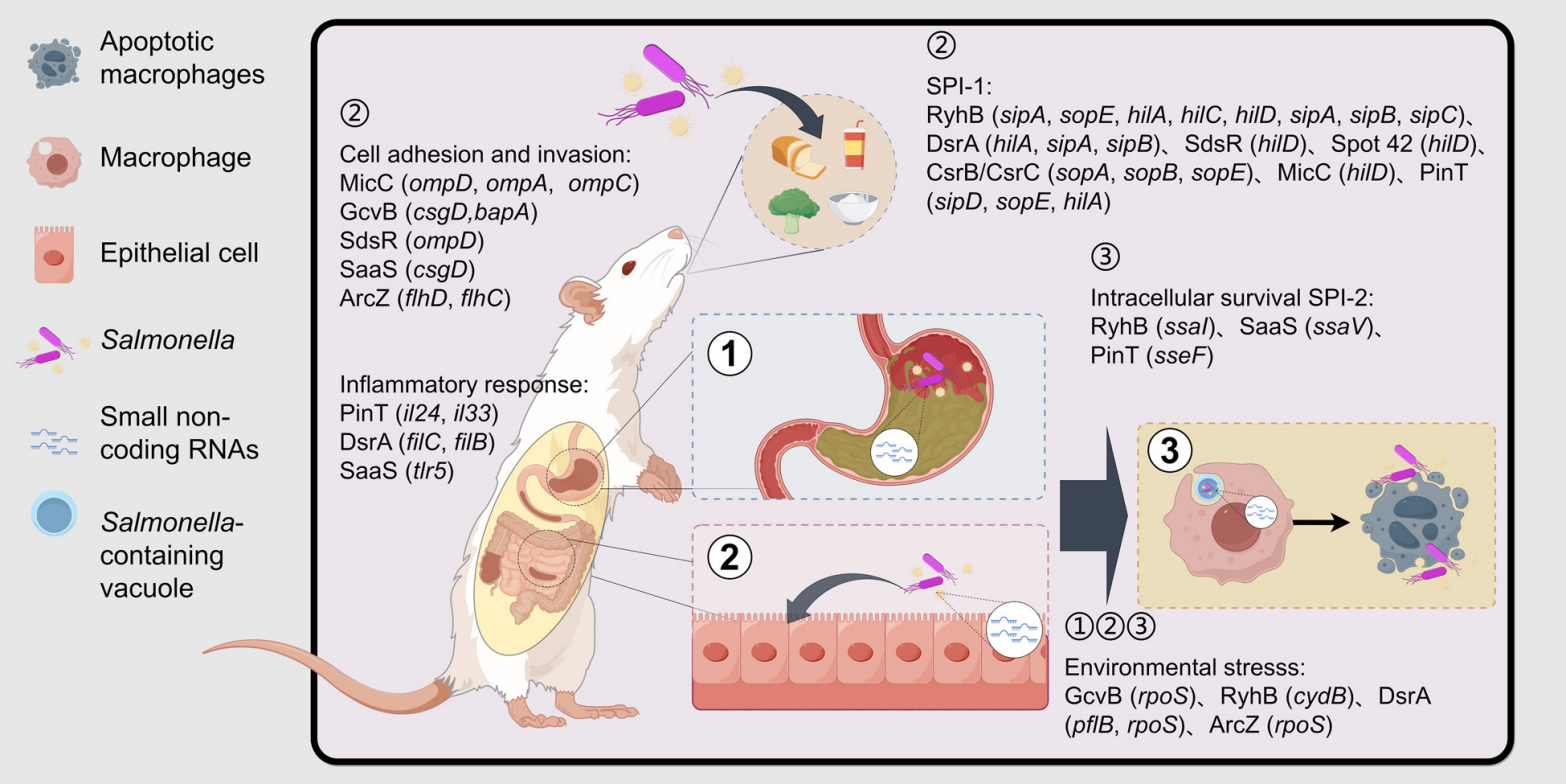
Associated processes involved in sRNAs after *Salmonella* invasion of the host. *Salmonella* is ingested by the host along with water and food and reaches the host’s stomach. Subsequently, it adheres to and invades intestinal epithelial cells with the assistance of biofilms, adhesins, and SPI-1 effector proteins. Finally, it survives within the SCV of macrophages with the help of SPI-2 effector factors. During the infection process, *Salmonella* needs to withstand a series of environmental stresses, including gastric acid, nutritional limitations in the intestine, and the antibacterial system of macrophages, and evade the host’s immune response to survive ultimately. sRNAs are involved in the regulation of environmental stress, epithelial cell adhesion and invasion, macrophage intracellular survival, and immunomodulation. The genes in the brackets are the key target genes of sRNA. (Created by Figdraw.).

*Salmonella* enters the host with food and water, and since the host stomach contains gastric acid, the bacteria are stimulated by the low PH environment and need to react to counteract this stimulus (Foster and Hall, 1990). This regulation enables the bacteria to stabilize their internal pH, allowing them to survive in the acidic environment. After passing through the stomach and entering the small intestine, *Salmonella* adheres to the intestinal epithelium using adhesins and invades the epithelium via bacterial-mediated endocytosis or through M cells (Shaji et al., 2023). Upon entering host cells, *Salmonella* utilizes its Type III secretion system (T3SS) to release proteins that interact with the host cells, aiding bacterial survival and replication. These interactions induce host cytoskeletal rearrangements, disrupt the normal epithelial brush border structure, and form membrane ruffles that encapsulate the bacteria in a large vesicle known as *Salmonella*-containing vacuoles (SCVs) (Chatterjee et al., 2023). Upon invading intestinal epithelial cells, an inflammatory response is triggered in the host, leading to symptoms such as diarrhea and fever. After invading the intestinal epithelium, *Salmonella* is phagocytosed by immune cells such as macrophages, dendritic cells, and neutrophils. From there, the bacteria can translocate to other organs, including the liver, spleen, and bone marrow, via the small intestinal lymphoid tissues, mesenteric lymph nodes, or through hematogenous spread (Shaji et al., 2023; Zhang et al., 2024). During this process, antimicrobial effectors, such as antimicrobial peptides, are produced within macrophages. However, *Salmonella* is able to survive and replicate within these cells by utilizing a large number of virulence factors that resist and evade the antimicrobial effects of macrophages, ultimately achieving systemic infection (Shaji et al., 2023). The infection pathway of *Salmonella* is shown in **Figure 1**.

*Salmonella* Pathogenicity Islands (SPIs) play a crucial role in the pathogenesis of *Salmonella*. Among these, *Salmonella* Pathogenicity Island 1 (SPI-1) was one of the first SPIs to be recognized and is primarily responsible for bacterial invasiveness and inflammation induction (Worley, 2023). SPI-1 encodes a T3SS that directs effector proteins into host cells, leading to alterations in host cell membrane structure and facilitating bacterial invasion (Chatterjee et al., 2023). Key effector proteins encoded by SPI-1, such as *sipA*, *sipB*, and *sopC*, promote bacterial entry into host cells via the T3SS (Azimi et al., 2020). *Salmonella* Pathogenicity Island 2 (SPI-2) is primarily responsible for bacterial replication and survival within phagocytes. Like SPI-1, SPI-2 also encodes a T3SS, but this system functions primarily after *Salmonella* has entered the host cell, helping the bacteria to survive and multiply within the host cell and avoid clearance by the host immune system (Schultz et al., 2021). Effector proteins encoded by SPI-2, such as *sseF* and *sseG*, help maintain the stability of SCVs and regulate nutrient uptake (Schultz et al., 2021). In addition to SPI-1 and SPI-2, *Salmonella* possesses multiple other virulence islands that encode proteins for a variety of functions, including metal ion transport, metabolic pathways adapted to the host environment, cell adhesion, and evasion of the host immune system. For example:SPI-3 is associated with the acquisition of magnesium ions; SPI-4 mediates adhesion to gastrointestinal epithelium; SPI-5 triggers an inflammatory response; SPI-6 possesses antimicrobial activity and promotes intracellular survival in macrophages, as well as systemic infections; SPI-7 attenuates the innate immune response and helps bacteria resist the killing effects of phagocytes. Together, these virulence islands enable *Salmonella* to adapt to the host environment, evade the host immune system, acquire necessary nutrients, and promote bacterial proliferation and survival within the host (Worley, 2023).

## 3 Mechanisms of sRNAs function in *Salmonella*

**Figure 2:**
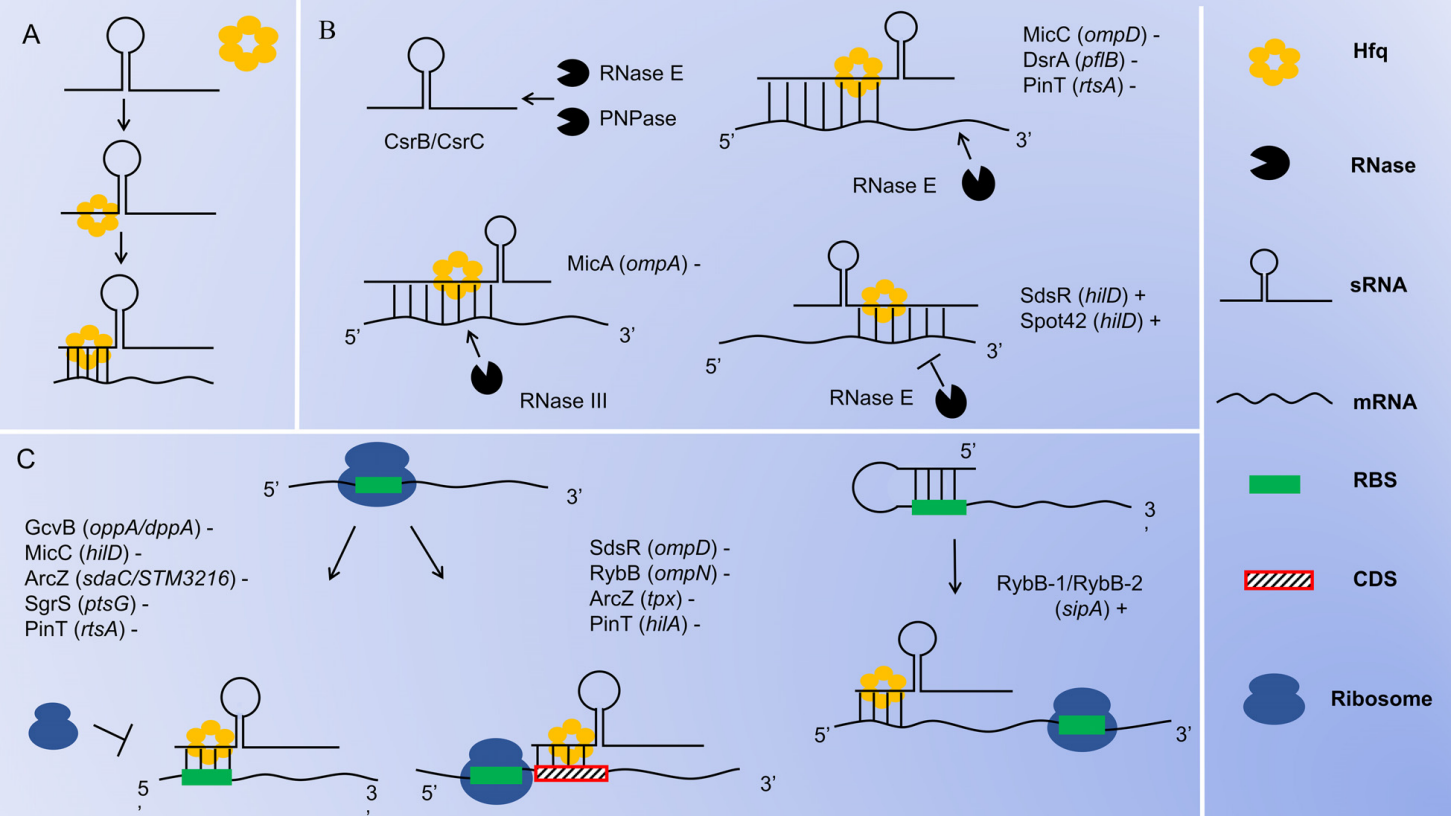
Mechanisms of sRNA Function in *Salmonella*. **(A)** Hfq stabilizes sRNA-mRNA complexes. **(B)** sRNA-mediated degradation or protection. RNase E and PNPase are capable of inducing the degradation of sRNAs CsrB/CsrC; The sRNAs bind to the 5’-UTR of the target mRNAs, recruiting RNase E to cleave and degrade the target mRNAs from the 3’ end; The sRNAs bind to the 5’-UTR of the target mRNAs, recruiting RNase III to cleave and degrade the target mRNAs at the double-stranded region; The sRNAs bind to the 3’-UTR of the target mRNAs, interfering with the cleavage of the target mRNAs by RNase E and promoting the translation of the mRNAs. **(C)** sRNA-mediated translational inhibition or activation. sRNAs block ribosome binding by directly attaching to the RBS in the 5’-UTR of target mRNAs, inhibiting translation initiation; sRNAs prevent ribosomal translation by binding to the CDS region of the target mRNAs; sRNAs bind to the 5’-UTR of the target mRNAs, unwind its secondary structure and expose the RBS, which promotes the binding of ribosomes to the RBS and facilitates the initiation of translation. The genes in the brackets are the target genes of sRNAs. The “+” or “-” indicates the up-regulation or down-regulation of the target genes by the sRNAs.

sRNAs are non-coding RNA molecules that are transcribed but typically do not encode proteins. They can form stable secondary structures after transcription. Based on the functions and modes of action, sRNAs can be categorized into three main groups: sRNAs with housekeeping functions, sRNAs that bind to target proteins and sRNAs that bind to target mRNAs through base pairing. Most of the currently known sRNAs belong to the third category. sRNAs can also be divided into cis- and trans-encoded sRNAs based on their base pairing patterns (Meng et al., 2022). Cis-encoded sRNAs are transcribed from the antisense strand of their regulatory target genes and bind to their corresponding mRNA targets in a partially complementary manner, leading to transcription termination or translation initiation (Aiba, 2007). Trans-encoded sRNAs, on the other hand, are typically produced by independent transcription from intergenic regions of the chromosome or by processing of the 5’ or 3’ untranslated regions (UTRs) of mRNAs. They interact with multiple mRNA targets through incomplete base complementation, inhibiting or facilitating translation initiation and accelerating or mitigating the degradation of target mRNAs (King et al., 2022). The pairing of many trans-encoded sRNAs with target mRNAs often requires the involvement of the RNA chaperone protein (Hfq) to enhance their interaction, and homologous Hfq has been found in a wide range of bacteria (Jørgensen et al., 2020). Hfq proteins are stable, cyclic hexameric structures composed of six identical subunits that can stabilize and protect RNAs as well as promote inter-pairing between RNAs (Watkins and Arya, 2023), As shown in **Figure 2**.

In *Salmonella*, most identified sRNAs block translation by directly binding to the ribosome binding site (RBS) in the 5’ UTR of target mRNAs, thereby down-regulating the expression of related genes. For example, GcvB blocks the translation of *oppA* and *dppA* mRNAs (Sharma et al., 2007); MicC blocks the translation of *hilD* mRNA (Cakar et al., 2022); ArcZ blocks the translation of *sdaC* and *STM3216* mRNAs (Papenfort et al., 2009); SgrS blocks the translation of *ptsG* mRNA (Bobrovskyy and Vanderpool, 2014); and PinT blocks the translation of *rtsA* mRNA (Kim et al., 2019b). This process is illustrated in **Figure 2**. Alternatively, some sRNAs inhibit translation initiation by binding to the coding sequence (CDS) region of target mRNAs. For instance, RybB inhibits the translation initiation of *ompN* mRNA (Bouvier et al., 2008); SdsR inhibits the translation initiation of *ompD* mRNA (Fröhlich et al., 2012); ArcZ inhibits the translation initiation of *tpx* mRNA (Papenfort et al., 2009); and PinT inhibits the translation initiation of *hilA* mRNA (Kim et al., 2019b). This process is illustrated in **Figure 2**. In contrast, some sRNAs activate translation by binding to the 5’ UTR of target mRNAs. Typically, these target mRNAs contain intrinsic secondary structures within their 5’ UTR that inhibit ribosome binding. Therefore, when sRNAs bind to the inhibitory sequences within the 5’ UTR, the RBS becomes accessible for ribosome binding, promoting translation initiation, and thus up-regulating the expression of related genes (Lalaouna et al., 2013). For example, RyhB-1 and/or RyhB-2 bind to the 5’ UTR region of *sipA*, opening up the secondary structure to reveal the hidden RBS and enhancing *sipA* expression (Chen et al., 2021). This process is illustrated in **Figure 2**. Through this interaction with the target mRNAs, sRNAs achieve direct regulation of the target gene.

sRNAs can also mediate the decay of target mRNAs. Ribonucleases (RNases) are key regulators of RNA decay, and sRNAs pair with target mRNAs to trigger RNase-mediated degradation (Lalaouna et al., 2013). Most decay processes are carried out by RNase E. RNase E is a single-strand-specific endoribonuclease that can enter from the 3’ end of a double-stranded RNA structure, extend linearly along single-stranded regions, and cleave the target mRNAs at appropriate sites, thereby down-regulating the expression of related genes (Waters et al., 2017). For example, MicC, with the assistance of Hfq, recognizes *ompD* mRNA and induces RNase E-mediated cleavage downstream of *ompD* mRNA (Pfeiffer et al., 2009; Bandyra et al., 2024); The base pairing of DsrA with *pflB* mRNA recruiting RNase E to degrade *pflB* mRNA (Dong et al., 2022); The base pairing of PinT with *rtsA* mRNA recruiting RNase E to degrade *rtsA* mRNA (Kim et al., 2019b). This process is illustrated in **Figure 2**. Conversely, SdsR and Spot 42 target the 3’ UTR of *hilD* mRNA. These sRNAs stabilize *hilD* mRNA levels by interfering with RNase E-dependent mRNA degradation mechanisms, thus up-regulating the expression of *hilD* (Abdulla et al., 2023). This process is illustrated in **Figure 2**. Moreover, RNase E is essential for the processing of sRNAs derived from the 3’ UTR of mRNAs. Hfq assists in guiding RNase E to cleave stable 3’ fragments from various precursor RNAs, leading to the formation of functional mature sRNAs. One notable example of this process is the maturation of ArcZ, which is cleaved by RNase E (Chao et al., 2017). RNase III is an endoribonuclease with specificity for double-stranded RNA (Quendera et al., 2020). In *Salmonella*, MicA interacts with its target *ompA* mRNA to form a double-stranded RNA structure. RNase III then targets this double-stranded region and mediates its degradation, thereby down-regulating the expression of *ompA* (Viegas et al., 2011). As shown in **Figure 2**. sRNAs achieve indirect regulation of target genes by recruiting RNase E or RNase III to induce the decay of target mRNAs, or by interfering with RNase E to prevent the decay of target mRNAs. PNPase is an exoribonuclease that progressively degrades RNA. In *Salmonella*, both CsrB and CsrC are highly structured molecules. The decay process of CsrB and CsrC involves multiple endonucleolytic cleavages by RNase E, which are then followed by exonucleolytic degradation by PNPase. PNPase has been identified as a key factor in the decay of CsrB and CsrC sRNAs in *Salmonella* (Viegas et al., 2007). As shown in **Figure 2**.

## 4 Functions of *Salmonella* virulence-associated sRNAs

To further understand the functions of virulence-associated sRNAs in *Salmonella*, we have listed several sRNAs that have been extensively studied in recent years. These sRNAs can indirectly regulate bacterial virulence by participating in various physiological processes, such as the regulation of outer membrane proteins, iron metabolism, glucose metabolism, carbon source utilization, redox balance, and antibiotic resistance. Additionally, these sRNAs play crucial roles in different stages of *Salmonella* infection, including gastrointestinal stress, invasion of intestinal epithelial cells, survival within macrophages, and the regulation of SPI-1 and SPI-2 (as shown in **Figure 1**), to promote bacterial colonization and infection. These sRNAs interact with multiple targets, forming a complex regulatory network that is involved in *Salmonella* survival and pathogenesis. We have integrated the basic information of the sRNAs mentioned below into **Table 1**, including their names, sizes, encoding modes, target mRNAs, and functions.

**Table 1:** Confirmed sRNAs of *Salmonella* spp.

sRNA	Length(nt)	Categorization	Target mRNA(s)	Function	Virulence	References
GcvB	200	Trans-encoded	*rpoS; csgD; bapA*	Glycine metabolism; General stress; Biofilm formation; Bacterial virulence	Enhance bacterial virulence in mice	Miyakoshi et al., 2022; Urbanowski et al., 2000; Miyakoshi et al., 2015; Bak et al., 2014; Andreassen et al., 2018
RyhB-1 (RyhB/RfrA)	96	Trans-encoded	*sipA; sopE; hilD; sipB; ssaI*	Iron metabolism; Bacterial virulence; Anti-oxidative stress	Enhance the invasion of Caco-2 cells; Dampen the virulence within macrophages	Kim and Kwon, 2013; Kim, 2016; Chen et al., 2021; Peñaloza et al., 2021; Meng et al., 2023; Calderón et al., 2014b
RyhB-2 (IsrE/RfrB)	98	Trans-encoded				
DsrA	87	Trans-encoded	*rpoS; hns; hilA; sipA; pflB*	General stress; Bacterial virulence; Redox balance	Enhance bacterial virulence in mice	Lease et al., 1998; Lalaouna et al., 2015; Ryan et al., 2016; Dong et al., 2022
SdsR (RyeB)	104	Trans-encoded	*stpA; crp; tolC; ompD; hilD*	Antibiotic resistance; Bacterial virulence; Biofilm formation	Enhance bacterial virulence in mice	Melamed et al., 2016; Gupta et al., 2019; Gutierrez et al., 2013; Doetsch et al., 2011; Abdulla et al., 2023
Spot 42	109	Trans-encoded	*galK; srlA; fucI; nanC*	Secondary metabolism; Uptake and utilization of non-priority carbon sources; Bacterial virulence	Enhance bacterial virulence in mice	Bækkedal and Haugen, 2015; El Mouali et al., 2021; Beisel and Storz, 2011; Abdulla et al., 2023
SaaS	unknown	Trans-encoded	*ssaV*	Biofilm formation; Bacterial virulence	Enhance bacterial virulence in mice	Hu et al., 2020; Cai et al., 2023a, 2023
ArcZ	121	Trans-encoded	*rpoS; cyaR; csgD; hilD; flhDC*	Biofilm formation; Bacterial virulence	Down-regulate the virulence factors, but the specific virulence has not been determined	Chao et al., 2017; Mika and Hengge, 2014; Kim et al., 2019a
CsrB/C	363/244	Trans-encoded	*SopA; SopB; SopE* *Pdu; cbi-cob*	Metabolism; Bacterial virulence	Up-regulate or down-regulate virulence-related genes, but the specific virulence has not been determined	Viegas et al., 2007; Nava-Galeana et al., 2023; El Mouali et al., 2021
MicC	109	Trans-encoded	*ompD; ompA; ompC; hilD*	Acid tolerance response; Bacterial virulence; Outer membrane protein formation	Dampen the virulence in mice and chickens	Pfeiffer et al., 2009; Wang et al., 2020; Meng et al., 2021; Cakar et al., 2022
PinT	80	Trans-encoded	*sopD; sipD; sopE; sseF; sipB; sopD2; hilA; rtsA; ssrB; steC*	Bacterial virulence; Host immune response	Dampen the virulence in mice	Westermann et al., 2016; Kim et al., 2019b; Correia Santos et al., 2021
SgrS	227	Trans-encoded	*sopD*	Encoding SgrT protein; Carbon metabolism; Bacterial virulence	Down-regulate the virulence factor, but the specific virulence has not been determined	Bobrovskyy and Vanderpool, 2014; Papenfort et al., 2012; Baek and Yoon, 2023

### 4.1 GcvB

GcvB is a highly conserved sRNA found in various bacterial families, including *Enterobacteriaceae*, *Actinobacteriaceae*, *Pasteurellaceae*, *Photobacteriaceae*, and *Vibrionaceae* (Ju et al., 2021; Liu et al., 2022). Approximately 200 nt in length, GcvB is a trans-encoded sRNA that requires the chaperone protein Hfq for its auxiliary actions (Miyakoshi et al., 2022). GcvB expression is closely linked to glycine metabolism, and it can activate the transcription of GcvB when glycine levels are high (Urbanowski et al., 2000; Ernst and Downs, 2016). The target genes of GcvB encode products that are primarily amino acid transport proteins, particularly the ABC transporter proteins Dpp and Opp (Miyakoshi et al., 2015). ABC transporters are essential membrane transport systems that play a critical role in bacterial survival, virulence, and pathogenicity (Davidson et al., 2008). In addition to its role in amino acid transport, GcvB is involved in various physiological processes, including bacterial biofilm formation, acid tolerance responses, and oxidative stress responses. The *rpoS* gene encodes the σ subunit of RNA polymerase, which regulates the transcription of genes involved in general stress responses in *Escherichia coli* (Metaane et al., 2022). Studies have shown that GcvB positively regulates the *rpoS* gene post-transcriptionally, although the exact mechanism of this regulation remains to be elucidated (Bak et al., 2014). *csgD* is a transcriptional regulator of the *csgBAC* operon, which encodes curli fibrillar proteins. These proteins are major components of the extracellular matrix in *Escherichia coli* and *Salmonella* and are crucial for bacterial adhesion and biofilm formation (Long et al., 2024). It has been demonstrated that GcvB down-regulates the expression of the *csgD* mRNA, thereby controlling the production of curli fibrils (Andreassen et al., 2018). Research by Ma et al. (2024) has shown that the deletion of GcvB in *Salmonella* Typhimurium significantly enhances the bacterium’s biofilm formation ability and up-regulates the mRNA expression of the biofilm-related protein BapA. Additionally, the virulence of the GcvB deletion strain in mice was reduced compared to wild-type and complementary strains, leading to an increased survival rate of the mice. This indicates that GcvB promotes the virulence of *Salmonella* Typhimurium. The oxidative stress resistance of the GcvB deletion strain was also significantly enhanced. These findings suggest that GcvB plays a regulatory role in *Salmonella* Typhimurium biofilm formation, oxidative stress resistance, and virulence.

### 4.2 RyhB

RyhB was initially discovered in *Escherichia coli* and is approximately 90 nt in length (Massé and Gottesman, 2002). Subsequently, two homologous and functionally similar sRNAs were identified in *Salmonella*: RyhB-1 (also known as RyhB or RfrA) and RyhB-2 (also known as IsrE or RfrB), with lengths of 96 nt and 98 nt, respectively (Kim and Kwon, 2013). In addition to regulating iron metabolism, *Salmonella* RyhB is involved in resistance to oxidative stress, acid resistance, bacterial motility, and host cell invasion and intracellular survival, among other life processes.

In *Escherichia coli*, iron acquisition and storage are controlled by the global iron uptake regulator Fur protein and the sRNA RyhB. The Fur protein can recognize the RyhB sequence in iron-rich environments and negatively regulate the expression of the RyhB (Porcheron and Dozois, 2015). When bacteria are in an iron-rich environment, Fur binds to Fe^2+^ and forms the Fur-Fe^2+^ complex, which binds to the Fur box of RyhB, inhibiting its transcription. This leads to the derepression of iron storage-related proteins and the decreased expression of iron uptake genes activated by RyhB, thus increasing intracellular free iron storage and reducing extracellular iron uptake functions. Conversely, when cells are iron-deficient, Fur dissociates from Fe^2+^, lifting the inhibition on RyhB. Under low iron conditions, RyhB is abundantly expressed and reduces the stability and translational efficiency of mRNAs encoding nonessential iron-sulfur proteins and iron storage proteins (Kim, 2016). This regulation of iron metabolism by RyhB ensures that iron is taken up and used in essential pathways, playing a crucial role in maintaining intracellular iron homeostasis.

Moreover, RyhB can regulate gene expression by incomplete base pairing with target mRNAs, promoting *Salmonella* invasiveness (Malabirade et al., 2018). Specifically, *Salmonella* enterica RyhB-1 and RyhB-2 enhance the invasion of Caco-2 cells by up-regulating the expression of T3SS effector proteins like SipA and SopE under simulated intestinal conditions, thereby promoting the bacterial virulence (Chen et al., 2021). When *Salmonella* invades macrophages, it faces harsh conditions, including acidification, oxidative stress, and nutrient deficiencies. Peñaloza et al. (2021) found that in a model of *Salmonella* Typhimurium infection of mouse macrophage RAW264.7, deletion of RyhB-1 and/or RyhB-2 resulted in increased intracellular survival and accelerated intracellular replication within the macrophage. These mutant strains exhibited higher intracellular ATP levels and lower NAD/NADH ratios compared to the wild-type. RyhB-1 and RyhB-2 also inhibit *Salmonella* virulence within RAW264.7 by down-regulating transcriptional regulators such as *hilA*, *hilC*, and *hilD*, as well as SPI-1-related genes encoding effector proteins like *sipA*, *sipB*, and *sipC*. Meng et al. (2023) found that deletion of RyhB-1 and/or RyhB-2 similarly increased the survival of *Salmonella enteritidis* in HD11 cells through a model of *Salmonella enteritidis* infection in chicken macrophage HD11 cells. Additionally, RyhB-1 negatively regulates the SPI-2 gene, and both RyhB-1 and RyhB-2 directly interact with the *ssaI* CDS region through an incomplete complementary base-pairing mechanism, down-regulating *ssaI* expression to inhibit its mRNA translation, thereby inhibiting the virulence of *Salmonella enteritidis* within HD11 cells. Phagocytes have two important antimicrobial systems: the Phagocyte Oxidase (PHOX) pathway and the inducible Nitric Oxide Synthase (iNOS) pathway. These pathways produce Reactive Oxygen Species (ROS) and Reactive Nitrogen Species (RNS), respectively. ROS plays a crucial role in the early response to bacterial infection, while RNS restricts bacterial survival within host cells (Meng et al., 2022). *Salmonella* Typhimurium RyhB-1 and RyhB-2 are highly induced by nitrosoglutathione (S-nitrosoglutathione, GSNO), a nitric oxide donor compound used to induce nitrosative stress in macrophages, which is an extremely potent antibacterial agent (Calderón et al., 2014a). Significant up-regulation of RyhB-1 and RyhB-2 expression in *Salmonella* Typhimurium after H_2_O_2_ treatment leads to increased intracellular ROS levels, helping the bacteria resist oxidative stress (Calderón et al., 2014b).

### 4.3 DsrA

DsrA is a trans-encoded sRNA that is 87 nt in length (Lease et al., 1998). It tends to accumulate in large quantities at low temperatures (Repoila and Gottesman, 2001). DsrA regulates the expression of the *hns* and *rpoS* genes. *rpoS* encodes a sigma factor that responds to general stress and is highly conserved in *Escherichia coli*, *Salmonella*, and other related enteric bacteria. When bacteria encounter harsh conditions, they need to integrate multiple stress signals and initiate appropriate cellular responses to survive. *rpoS* is a major regulator of the general stress response (Jones et al., 2006). DsrA activates the translation of *rpoS* by base pairing with the 5’ UTR of *rpoS* mRNA. On the other hand, *hns* encodes the H-NS protein, a major repressor and silencer of numerous genes. The expression of DsrA triggers an increase in the turnover of *hns* mRNA, leading to a decrease in the amount of H-NS protein in the cells (Lalaouna et al., 2015).

The known functions of DsrA are primarily focused on its role in the acid tolerance response (ATR). *Salmonella* are exposed to various stress conditions during pathogenesis, with acid stress being one of the main host defense mechanisms. This acid stress environment is typically encountered in the host’s stomach and in phagocytic and non-phagocytic cells containing SCVs (Allam et al., 2012). Ryan et al. (2016) found that DsrA was induced threefold under conditions of pH 3.1 compared to normal conditions. Homozygous mutants lacking DsrA (ΔDsrA) exhibited lower viability under ATR, along with a 1.8-fold up-regulation of the *hns* gene in ΔDsrA compared to the wild strain. Additionally, the expression of invasion-associated SPI-1 effectors such as *hilA*, *sipA*, and *sipB* was down-regulated in ΔDsrA, resulting in reduced motility, weak adhesion, and impaired invasion efficacy, indicating that DsrA can promote the virulence of the bacteria. Furthermore, ΔDsrA was unable to induce intestinal inflammation in C57BL/6 mice 72 hours post-infection, which may be related to a significant reduction in the expression of flagellin structural genes *fliC* and *fliB* in ΔDsrA, considered key pro-inflammatory determinants.

Dong et al. (2022) found that genes involved in glycolysis, pyruvate metabolism, the tricarboxylic acid (TCA) cycle, and NADH-dependent respiration showed significantly different expression patterns between the wild strain of *Salmonella* Typhimurium and the DsrA deletion mutant (ΔDsrA), both before and after H_2_O_2_ treatment, as revealed by RNA-Seq. This suggests that DsrA plays a crucial role in regulating central carbon metabolism (CCM) and NAD(H) homeostasis in *Salmonella* Typhimurium. Target gene prediction and experimental validation revealed that DsrA down-regulates the expression of *pflB*, which encodes pyruvate-formate lyase. Additionally, the NAD/NADH ratio in the *pflB*-DsrA co-overexpression strain (WT-p*pflB*-pDsrA) was significantly lower than that in the *pflB* overexpression strain (WT-p*pflB*), suggesting that the inhibition of *pflB* by DsrA significantly affects the redox homeostasis of *Salmonella* Typhimurium. Changes in redox status are often closely related to alterations in bacterial metabolic pathways, activation of stress responses or expression of virulence factors.

### 4.4 SdsR(RyeB)

SdsR is a member of the stationary phase sigma factor (σS) regulator and is one of the most conserved sRNAs in *Enterobacteriaceae* (Ryan et al., 2017). It is present in *Escherichia coli*, *Salmonella*, and other enterobacteria. SdsR binds to target mRNAs through partial base complementary pairing (Melamed et al., 2016), with the binding region often consisting of six to eight adjacent bases (Bouvier et al., 2008). Hfq protects SdsR from degradation by cytosolic ribonucleases and promotes trans base pairing with multiple targets, such as *mutS* and *tolC* in *Escherichia coli* and *stpA*, *crp*, *tolC*, and *ompD* in *Salmonella* (Gupta et al., 2019).

Previous studies have found that SdsR can affect the translation of membrane proteins (Fröhlich et al., 2016), antibiotic susceptibility (Gutierrez et al., 2013), survival time during the stationary phase (Monteiro et al., 2012), and biofilm formation (Doetsch et al., 2011). SdsR also down-regulates the expression of *tolC*, a gene encoding an outer membrane protein component of drug-resistant efflux pumps, thereby reducing bacterial resistance to new antibiotics and crystal violet (Parker and Gottesman, 2016). In recent years, a study by Abdulla et al. (2023) found that the 3’ UTR of *hilD* renders *hilD* mRNA susceptible to RNase E-dependent degradation. However, SdsR alters the accessibility of *hilD* mRNA to RNase E cleavage by targeting the binding site within the first 90 nt of the 3’ UTR, thereby up-regulating the expression of *hilD* and SPI-1 genes. The transcriptional regulator *hilD* is a major regulatory element for SPI-1 virulence gene expression, and the regulatory effect of SdsR on *hilD* is essential for *Salmonella* invasion. SdsR accumulates in large quantities during the stationary phase, osmotic shock, and heat shock, and its expression is negatively correlated with that of the sRNA SraC (RyeA). The sRNA homologs of SdsR and SraC are located in the intergenic region (IGR) between the *pphA* and *yebY* genes, with opposite polarity (Gupta et al., 2019). Unlike SdsR, SraC does not depend on the regulation of Hfq and σ factors and is less conserved (Gupta et al., 2020). Consequently, SraC has been rarely studied in *Salmonella*. SraC is 270 nt long, and its internal fragment is fully complementary to the 104 nt long SdsR.

### 4.5 Spot 42

Spot 42 is a 109 nt sRNA specifically found in γ-proteobacteria, including *Escherichia coli* and *Salmonella* (Bækkedal and Haugen, 2015). The cyclic adenosine monophosphate receptor protein (CRP) is a transcription factor that becomes active upon binding to the intracellular second messenger cyclic adenosine monophosphate (cAMP). The interaction of CRP-cAMP with DNA results in the activation or inhibition of its target genes (El Mouali et al., 2021). Spot 42 is highly expressed when bacteria are grown under glucose conditions, while its transcription is inhibited by cAMP-CRP when the bacteria are grown on non-preferred carbon sources (Bækkedal and Haugen, 2015). Spot 42 can be divided into two functional domains. The 5’ part comprises three distinct single-stranded regions involved in base pairing with mRNA targets, while the 3’ region contains the Hfq binding module, consisting of two stem-loop structures (Stenum and Holmqvist, 2022). Spot 42 RNA has been shown to down-regulate several targets, including *galK*, *srlA*, *fucI*, and *nanC*, which encode proteins involved in secondary metabolism and the uptake and utilization of non-preferred carbon sources (Beisel and Storz, 2011). Aoyama et al. (2022) found that Spot 42 encodes a 15-amino acid protein, SpfP, which plays a role in metabolic inhibition. This indicates that Spot 42 is a dual-function sRNA, affecting both carbon source utilization through its base-pairing activity and via the small protein it encodes. Additionally, Abdulla et al. (2023) demonstrated that two sRNAs, SdsR and Spot 42, regulate SPI-1 by targeting different regions of the *hilD* mRNA 3’ UTR. *Salmonella* mutants lacking both SdsR and Spot 42 showed reduced virulence in mouse infection models, indicating that SdsR and Spot 42 promote bacterial virulence. Unlike the binding site of SdsR on *hilD*, Spot 42 targets a region of *hilD* closer to the terminator.

### 4.6 SaaS

SaaS is a newly identified regulatory factor screened from meat-source *Salmonella* enteritidis in recent years. Hu et al. (2020) investigated the transcription and sRNA expression of *Salmonella enteritidis* exposed to meat thawing loss broth (MTLB) during biofilm formation. Through RNA-Seq, they identified 19 candidate sRNAs involved in biofilm regulation. Preliminary findings suggest that SaaS is associated with the biofilm formation of *Salmonella enteritidis*, which is closely linked to bacterial colonization. This implies a potential connection between SaaS and the virulence of *Salmonella enteritidis*. Cai et al. (2023a) studied the role of SaaS in the virulence phenotype of *Salmonella enteritidis* by constructing SaaS mutant and complementary strains. They found that SaaS is activated in a simulated intestinal environment (SIE) and can directly target the mRNA of the virulence protein SsaV, up-regulating the expression of *ssaV*. Experiments using human colorectal adenocarcinoma cells (Caco-2) and mouse macrophages (RAW264.7) showed that SaaS promotes the invasion and destruction of epithelial cells by *Salmonella enteritidis* and inhibits the overgrowth and destruction of macrophages. Additionally, BALB/c mouse models demonstrated that the removal of SaaS significantly reduced mortality, mitigated pathophysiological deterioration, and decreased the spread of bacteria to the systemic circulation and systemic inflammation. SaaS also promoted bacterial colonization in the cecum and colon of the BALB/c mouse model. Furthermore, Cai et al. (2023b) found that SaaS enhances damage to the mucosal barrier by affecting the expression of antibacterial products, reducing the number of goblet cells, inhibiting the expression of mucin genes, and ultimately decreasing the thickness of the mucosal layer. High-throughput 16S rRNA gene sequencing revealed that SaaS alters gut balance by depleting beneficial gut microbiota and increasing harmful ones. ELISA and Western blot analyses confirmed that SaaS regulates intestinal inflammation by continuously activating the P38-JNK-ERK MAPK signaling pathway, achieving immune escape in the initial infection stage and enhancing pathogenesis in later stages. These studies all indicate that SaaS promotes the virulence of *Salmonella enteritidis*.

### 4.7 ArcZ

ArcZ is a conserved trans-acting sRNA in *Enterobacteria* that relies on Hfq and is cleaved by RNase E into a processed form of 55 to 60 nt. This processed form is highly conserved for controlling the expression of target mRNAs (Chao et al., 2017). High oxygen levels can induce the expression of ArcZ, which peaks during the stationary growth phase. This regulation is mediated by the oxygen-responsive two-component system ArcA/ArcB, which restricts the transcription of ArcZ under low oxygen conditions (Brown et al., 2022). ArcZ can control multiple targets and influences stress response, motility, and virulence by regulating major regulators such as *flhDC* and *rpoS*. Recent RL-SEQ data suggest that ArcZ may interact directly with more than 10% of mRNAs in the *Escherichia coli* and *Salmonella* genomes, making ArcZ one of the largest target regulators among sRNAs (Melamed et al., 2016; Liu et al., 2023). In *Escherichia coli*, the general stress response mediated by RpoS has been extensively studied, and the first clear target of ArcZ in *Escherichia coli* is *rpoS* mRNA (Mandin and Gottesman, 2010; Battesti et al., 2011). ArcZ can directly act on *rpoS* mRNA, up-regulating the expression of the *rpoS* gene. Another sRNA, CyaR, can interact with *rpoS* mRNA and down-regulate *rpoS* mRNA expression (Kim and Lee, 2020). ArcZ can directly interact with CyaR and inhibit its function by degrading CyaR through RNase E (Kim and Lee, 2020), while CyaR does not affect the activity of ArcZ (Iosub et al., 2020). ArcZ can control the biofilm formation of *Salmonella* Typhimurium by regulating the transcriptional factor CsgD (Mika and Hengge, 2014). CsgD regulates multiple genes responsible for the assembly, transport, and synthesis of curli, which are crucial for biofilm formation. Recently, Kim et al. (2019a) found that in *Salmonella* Typhimurium, two sRNAs, FnrS and ArcZ, directly affect the activation of the SPI1 T3SS by regulating *hilD* translation. ArcZ and the two-component system FnrS inhibit the translation of *hilD* mRNA through direct interaction with the mRNA of the virulence gene *hilD*, thereby down-regulating the expression of hilD and hilA. Under aerobic conditions, ArcZ is expressed and inhibits *hilD* translation. Under hypoxic conditions, the two-component Fnr system activates the transcription of FnrS, which in turn inhibits *hilD* translation. This regulatory network allows the T3SS genes to be expressed most efficiently when exposed to fluctuating oxygen levels.

### 4.8 CsrB/CsrC

The sequence-specific RNA-binding protein CsrA is a global regulator that controls the expression of SPI-1 and SPI-2 by directly inhibiting *hilD* translation initiation and down-regulating the expression of *hilD*. Additionally, CsrA regulates the expression of genes encoding metabolic regulators and biofilm formation in *Salmonella*. It plays a crucial role in modulating the pathogenicity, metabolism, biofilm formation, and motility of bacteria in response to changes in nutritional conditions. The activity of CsrA is regulated by two sRNAs, CsrB and CsrC, which sequester and antagonize the CsrA protein (Potts et al., 2019). The BarA/SirA two-component system induces the expression of CsrB and CsrC. Together, the BarA/SirA and Csr global regulatory systems control a wide range of cellular processes. Nava-Galeana et al. (2023) found through proteomic analysis that in *Salmonella* Typhimurium, the BarA/SirA and CsrB/CsrC systems up-regulate the expression of SPI-1-related genes such as *sopA*, *sopB* and *sopE* in a cascade manner, and down-regulate the genes expressed in the intestinal lumen of *Salmonella*, such as *pdu* and *cbi-cob*. Additionally, a study by El Mouali et al. (2021) found that in *Salmonella* enteritidis, the central metabolic regulator CRP-cAMP can regulate CsrB and CsrC to varying degrees in a growth-stage-dependent manner.

### 4.9 MicC

MicC is capable of binding to the mRNA of the outer membrane protein OmpD and indirectly down-regulate its expression in *Salmonella* Typhimurium by accelerating the decay of *ompD* mRNA through RNase E-dependent mechanisms (Pfeiffer et al., 2009). Wang et al. (2020) exposed five strains of *Salmonella* to acid stress and found that acid exposure induced high levels of MicC expression. Meng et al. (2021) constructed a mutant and a replacement strain of *Salmonella enteritidis* MicC using a λRed recombination system. qRT-PCR results showed that MicC down-regulated the expression of *ompA* and *ompC*. Additionally, challenge experiments using 6-week-old BALB/c mice and 1-day-old chickens demonstrated that MicC may inhibit the virulence of *Salmonella enteritidis* to mice and chickens by down-regulating the expression of outer membrane protein genes. Cakar et al. (2022) found that MicC prevents the translation of *hilD* mRNA by base pairing near the ribosome binding site, and down-regulates the expression of *hilD*. MicC is activated by the transcription factor SlyA, and SlyA enters the SPI-1 regulatory network exclusively through MicC. The transcription of MicC is negatively regulated by the OmpR/EnvZ two-component system, but this regulation is dependent on SlyA. Deletion mutants of MicC were more competitive in the small intestines of BALB/c mice compared to wild-type strains, indicating that MicC inhibits bacterial virulence during infection.

### 4.10 PinT

PinT is an 80 nt long sRNA derived from a horizontally acquired *Salmonella*-specific site that also encodes the co-activating protein RtsA of the invasive gene (Westermann et al., 2016). Studies by Westermann et al. (2016) identified PhoP-activated PinT, which increases 100-fold during infection after entry into host cells. PinT can affect the expression of invasion-related effectors and virulence genes required for intracellular survival. Specifically, the expression of SPI-1 invasion-related genes (such as *sipD and sopE*) decreases, while the expression of SPI-2 genes that promote intracellular survival (such as *sseF*) increases. Additionally, NF-κB-related immune genes are strongly activated in the invading host cells. The expression of the SPI-1 and SPI-2 T3SS is no longer required after *Salmonella* invades intestinal epithelial cells or during intracellular replication in macrophages. Kim et al. (2019b) found that PhoPQ-regulated sRNA PinT promotes the effective down-regulation of the SPI-1 T3SS by preventing the translation of *hilA* and *rtsA*. PinT base-pairs with the mRNAs of hilA and rtsA, inhibits the initiation of *hilA* translation, and directly down-regulates the expression of *hilA*. Meanwhile, it also induces the degradation of *rtsA* transcripts and indirectly down-regulates the expression of *rtsA*. PinT also indirectly down-regulates the expression of *fliZ*, a post-translational regulator of HilD, and directly inhibits the translation of SsrB, a major regulator of SPI-2 T3SS, thus down-regulating the expression of *ssrB*. *In vivo* mouse competition tests have shown that PinT controls a series of virulence genes at the post-transcriptional level, and PinT is able to inhibit the virulence of bacteria to mice. Additionally, PinT is capable of inhibiting the mRNA of the effector kinase SteC in *Salmonella* Typhimurium, thereby down-regulating the expression of *steC* and ultimately delaying actin rearrangement in infected host cells (Correia Santos et al., 2021).

### 4.11 SgrS

SgrS is a 227 nt Hfq-bound sRNA that is widely present in γ-proteobacteria. Its 5’ terminal also encodes a small 43-amino acid protein called SgrT, which inhibits the activity of major glucose transporters. SgrS has two distinct functions: it base-pairs with *ptsG* mRNA, which encodes the glucose transporter EIICB^Glc^, to inhibit its translation, and it also translates the small protein SgrT from its own open reading frame (ORF). These functions operate through independent regulatory mechanisms, both of which can reduce the accumulation of phosphorylated sugars, thereby relieving stress and promoting growth (Bobrovskyy and Vanderpool, 2014). SgrS can pair with the mRNA of the virulence gene *sopD* and inhibit its translation, thus down-regulating the expression of *sopD* (Papenfort et al., 2012). c-di-GMP is a central signaling molecule that determines the transition between motile and sessile lifestyles in many bacteria. At high concentrations, c-di-GMP stimulates the formation of biofilms, while at low concentrations, it leads to the dispersion and planktonic state of biofilms. In recent years, Baek and Yoon (2023) have studied the potential of c-di-GMP to alter the carbon metabolic pathway of *Salmonella* Typhimurium. They found that the loss of SgrS alleviates the down-regulation of the carbohydrate phosphotransferase system (PTS) genes by c-di-GMP. These results indicate that SgrS mediates the negative effects of c-di-GMP on PTS regulation.

## 5 Conclusion and prospect

Base pairing between sRNA and target mRNA can lead to mRNA translation inhibition or activation, as well as promote mRNA stabilization or degradation. This interaction forms a complex regulatory network in *Salmonella*, playing a crucial role in bacterial adaptation, virulence, and pathogenesis. *Salmonella* is an enteric pathogen and intracellular parasite. When the bacteria enter the host through contaminated water and food, they encounter various pressures and stresses in the host’s gastrointestinal tract and macrophages. sRNAs such as GcvB, RyhB, DsrA, and ArcZ are involved in regulating the stress response, enabling the bacteria to adapt to environmental changes. Upon entering the intestine, the bacteria must adhere to and invade intestinal epithelial cells. Bacterial biofilm formation promotes adhesion to these cells, and sRNAs like GcvB, SdsR, SaaS, ArcZ, and CsrB/CsrC participate in regulating biofilm formation, thus enhancing adhesion to epithelial cells. Additionally, SPI-1 is a key virulence island involved in the adhesion and invasion of intestinal epithelial cells. sRNAs such as RyhB, DsrA, SdsR, Spot 42, SaaS, CsrB/CsrC, MicC, SgrS and PinT regulate the translation of SPI-1-related virulence gene mRNAs, thereby modulating their virulence. After *Salmonella* enters macrophages, it needs to survive and replicate within the SCV. SPI-2 plays a crucial role in the intracellular survival stage of the bacteria, and sRNAs such as RyhB, SaaS and PinT regulate the translation of SPI-2-related gene mRNAs, promoting bacterial survival within the cells (as shown in **Figure 1**). Moreover, sRNAs are involved in the regulation of outer membrane proteins, iron metabolism, glucose metabolism, carbon source utilization, redox balance, antibiotic resistance, and numerous other physiological processes.

In the past five years, numerous new advancements have been achieved in the research of sRNAs. In previous studies, although a large number of sRNAs were identified, only a few of them have been proven to be capable of regulating target genes and had their signal regulatory pathways elucidated. Recently, researchers have identified a novel sRNA named SaaS from meat-derived *Salmonella enteritidis*. SaaS can enhance bacterial virulence and continuously activate the P38-JNK-ERK MAPK signaling pathway to regulate intestinal inflammation, which represents a significant new discovery regarding the regulation of the interaction between bacteria and the host by sRNAs (Cai et al., 2023b). In addition, RyhB was initially discovered in *Escherichia coli*, and subsequently, its homologous counterparts RyhB-1 and RyhB-2 were identified in *Salmonella* (Kim and Kwon, 2013). The more well-known function of RyhB-1 and RyhB-2 is related to the uptake and utilization of iron (Porcheron and Dozois, 2015; Kim, 2016). In recent years, multiple researchers have found that RyhB-1 and RyhB-2 can promote the invasion of intestinal epithelial cells in the early stage of infection (within the intestine), while in the late stage of infection (inside macrophages), they can inhibit the virulence of bacteria in the macrophages of mice and chickens (Chen et al., 2021; Peñaloza et al., 2021; Meng et al., 2023). This stage-dependent regulation reflects the precise regulatory ability of sRNAs in complex infectious microenvironments. In previous studies on the mechanism of sRNA-mediated decay of target mRNAs, sRNAs typically targeted the 5’-UTR of mRNAs and recruited RNases to cleave the target mRNAs. However, recent research has revealed that SdsR and Spot42 can target the 3’-UTR of *hilD*, interfering with the degradation function of RNase E and thus up-regulating the expression of *hilD* (Abdulla et al., 2023). This new discovery has revealed a novel regulatory pathway of bacterial virulence and expanded our understanding of the regulatory mechanism of sRNAs on target mRNAs. However, despite the new breakthroughs achieved in the research progress of sRNAs in recent years, there are still some issues that need to be improved and resolved:

1. In *Salmonella*, most studies have focused on the single functions of sRNAs. For example, as previously mentioned, SgrS acts as an intermediary in the c-di-GMP-mediated down-regulation of PTS genes and may indirectly influence bacterial biofilm formation by regulating PTS systems (Baek and Yoon, 2023). In addition, some sRNAs listed in **Table 1** primarily regulate biofilm formation indirectly by modulating outer membrane proteins or *rpoS* mRNAs. Although these sRNAs are known to play important roles in biofilm formation, whether they also play crucial roles in lifestyle or infection phase transitions remains unclear.
2. Additionally, the function of sRNAs in the process of bacterial infection, especially in regulating the host immune response, remains to be fully elucidated. Many sRNAs have been found to be capable of influencing bacterial virulence. Apart from regulating the expression of virulence factors, it remains unclear whether they also participate in the relevant signaling pathways of immune regulation. Moreover, the specific molecular recognition and signal transduction mechanisms between sRNAs and immune cells are still not well-defined.
3. There are various sRNAs in bacteria, and there may be synergistic or antagonistic effects among them, jointly regulating the physiological functions of bacteria and their interactions with the host. However, most current studies only focus on the functions of individual sRNAs, and there is relatively little research on the impacts of the combined actions of multiple sRNAs on host pathogenesis. For instance, different sRNAs may regulate different virulence genes and cooperate with each other to influence the infection process of bacteria. Nevertheless, the mechanisms of such combined actions have not been fully revealed.
4. Currently, there are certain limitations in the research field of *Salmonella* sRNAs. From the perspective of serotypes, research mainly focuses on *Salmonella* Typhimurium and *Salmonella enteritidis*. In contrast, for numerous other serotypes of *Salmonella*, the research on sRNAs is rather scarce, making it difficult for us to comprehensively understand the functions and regulatory mechanisms of sRNAs within the entire genus of *Salmonella*. In terms of the selection of animal models, the types of hosts are relatively limited, mainly being mice and chickens. This limitation has led to a lack of understanding of the mechanisms of action of *Salmonella* sRNAs within other important human or animal hosts, as well as their impacts on the pathological processes of the hosts. This not only restricts our grasp of the universal laws of *Salmonella* infections but also poses challenges to the transformation and application of relevant research findings in different host backgrounds.

Therefore, the role of sRNAs warrants further attention. With the advancement of bioinformatics and the continuous progress and improvement of second-generation sequencing technology, more sRNAs and their specific functions will be discovered in the future, leading to a deeper understanding of bacterial survival and pathogen-host interactions. This review summarizes the regulatory roles of several sRNAs in *Salmonella*-host cell interactions that have been well studied in recent years, providing valuable insights and new research directions in the field. It is expected to have a significant impact on the research, diagnosis, control, and treatment of the disease. For example, in terms of disease diagnosis, sRNAs can serve as stage-specific markers of infection (such as the high expression of SaaS in the intestinal environment). Early diagnosis can be achieved by detecting the levels of sRNAs in clinical samples. In terms of disease control, targeting biofilm-related sRNAs (such as GcvB) can inhibit bacterial adhesion and colonization, reducing the risk of foodborne contamination. In terms of disease treatment, taking sRNAs that regulate virulence (such as SdsR) as targets, developing specific inhibitors to promote their degradation or suppress their functions can weaken the pathogenicity of *Salmonella*, making it easier for the body to eliminate the bacteria.
